# A novel ablation strategy for refractory atrial fibrillation based on the fractionated signal area in the atrial muscle

**DOI:** 10.1016/j.hrcr.2021.09.010

**Published:** 2021-09-24

**Authors:** Jun Hirokami, Kenichi Hiroshima, Michio Nagashima, Masato Fukunaga, Kengo Korai, Kenji Ando

**Affiliations:** Department of Cardiology, Kokura Memorial Hospital, Kitakyushu, Japan

**Keywords:** Atrial fibrillation, Radiofrequency ablation, Refractory atrial fibrillation, Fractionated signal, Non–pulmonary vein foci

## Introduction

Pulmonary vein isolation (PVI) for atrial fibrillation (AF) is a well-established treatment.^1^, ^2^, ^3^ Non–pulmonary vein (PV) trigger elimination can improve the recurrence rate after paroxysmal and persistent AF ablation.^4^^,^^5^ However, complete non-PV trigger elimination is challenging owing to difficulty in detecting the non-PV foci. We developed a novel visualization tool of the fractionated signal area in the atrial muscle (FAAM) map, which highlights the fractionated signal area using Lumipoint software in the ultra-high-density Rhythmia (Boston Scientific, Marlborough, MA) system and successfully identifies the non-PV foci.^6^ Herein, we report a novel ablation strategy, FAAM ablation, which is effective for refractory AF.

### What the FAAM map is

FAAM maps were created using Lumipoint software in the Rhythmia system on an offline workstation. Low-voltage areas were defined as areas with amplitudes between 0.1 and 0.5 mV, and no-voltage areas were defined as areas with amplitudes of <0.03 mV. A fractionated signal was defined as a waveform with 2 or more fragmented deflections during sinus rhythm or atrial pacing. The FAAM was defined as the area where fractionated signals could be detected in the atrium. FAAM maps were assigned by a peaks slider, which is an original parameter of Lumipoint. The peaks slider indicates the number of components of fractionated signals and ranges from 1 to 15. If a large peaks slider was set, Lumipoint highlighted a small area that only had high fractionated signals; the larger the peaks slider is, the smaller the FAAM is. The “Activation search” function of the Lumipoint software was applied to all atrial maps. This feature uses an adjustable time-of-interest period within the mapping window to highlight all electrocardiograms (ECGs) that show activity during the time-of-interest period. The “Complex Activation search” function highlights ECGs that show activity within the time-of-interest period and exhibit multiple fractionated signals. The “Complex Activation search” of Lumipoint highlighted the FAAM in the antrum.

## Case report

A 69-year-old man was diagnosed with symptomatic persistent AF 9 years ago. He had undergone PVI, superior vena cava (SVC) isolation, and ganglionated plexi ablation for persistent AF. Five years ago, he had recurrence of tachycardia by atrial tachycardia; thus he had undergone left atrial (LA) roof area ablation for atrial premature beat, and mitral isthmus and LA anterior line ablation for persistent atrial tachycardia. He had paroxysmal AF recurrence 1 year ago and frequent episodes of drug-resistant palpitation. He required a third catheter ablation session. Patients were permitted to this study participation upon admission to hospital.

At admission, 12-lead ECG showed sinus rhythm. A transthoracic echocardiogram showed a normal left ventricular ejection fraction of 65.4%, LA diameter of 35.5 mm, and LA volume index of 21.2. Laboratory data were as follows: brain natriuretic peptide, 15.0 pg/mL; thyroid-stimulating hormone, 2.87 μIU/mL; free thyroxine, 1.18 ng/dL; blood urea nitrogen, 14.7 mg/dL; and creatinine, 0.90 mg/dL. His past medical history was only significant for dyslipidemia: CHADS2 score 0 and CHA_2_DS_2_-VASc score 1 for age 65–74 years (congestive heart failure, hypertension, age ≥75 years, diabetes mellitus, history of stroke or transient ischemic attack, vascular disease, age 65–74, and sex). He was administered dabigatran etexilate methanesulfonate, 220 mg/day; bepridil hydrochloride hydrate, 100 mg/day; pitavastatin calcium hydrate, 2 mg/day; and verapamil hydrochloride, 40 mg pill-in-the-pocket.

During the procedure, a 20-pole catheter was inserted through the right jugular vein. The proximal electrodes were positioned along the SVC and crista terminalis, and the distal electrodes were placed in the coronary sinus. If AF continued, it was terminated using intracardiac defibrillation.

After performance of the standard Brockenbrough technique, mapping was performed using the ultra-high-density Rhythmia system during sinus rhythm with an Orion mapping catheter (Boston Scientific, Marlborough, MA). The following beat acceptance criteria were used: cycle length, propagation reference by the coronary sinus catheter distal and proximal electrodes, respiratory stability, motion stability of the Orion mapping catheter within 1 mm, and maximal distance of 2 mm between the electrode and anatomical shell.

**Figure 1:**
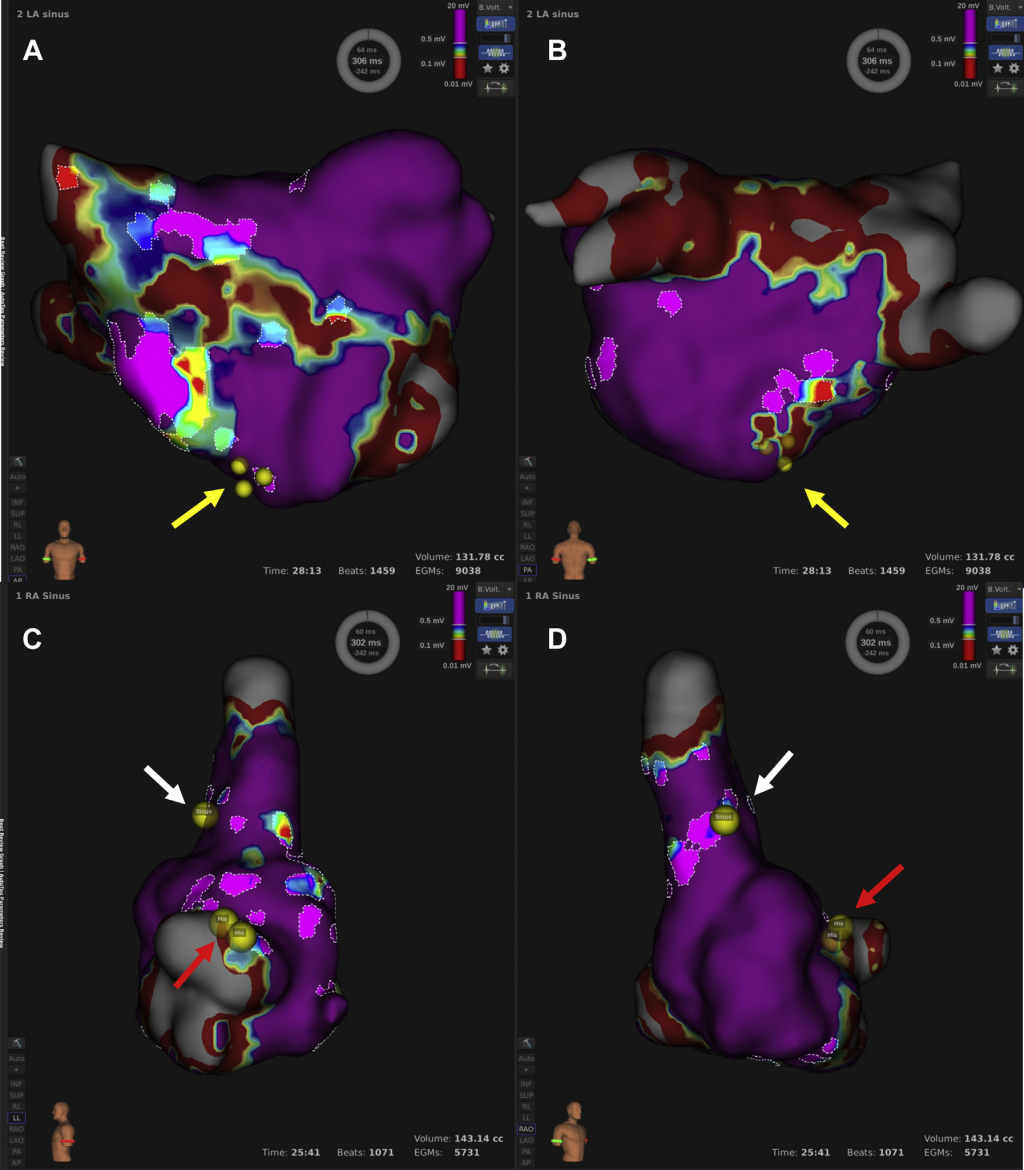
Voltage map and fractionated signal areas in atrial muscle (FAAM) map. **A, B:** Voltage map of left atrium showed broad low-voltage area in anterior wall and posterior roof wall. Non–pulmonary vein foci that could be detected (*yellow arrows*) were located within FAAM of peaks slider 6.0. **C, D:** Voltage map of right atrium showed almost-normal voltage. Sinus node (*white arrows*) and atrioventricular node (*red arrows*) were located near or within FAAM of peaks slider 5.0.

In this procedure, all PVs and the SVC were isolated. The low-voltage line was in LA anterior from mitral to right superior PV. Scar zones were in the LA roof area (Figure 1). We analyzed non-PV triggers with electrode catheters by administering a bolus injection of adenosine triphosphate (ATP; 60 mg) during a continuous infusion of isoproterenol (ISP; 10 μg/kg/min). However, detection of all non-PV triggers was difficult because of multiple spontaneous ectopic beats and induction of incessant AF. We previously reported FAAM is related to non-PV foci.^6^ Therefore, we decided to ablate the FAAM.

**Figure 2:**
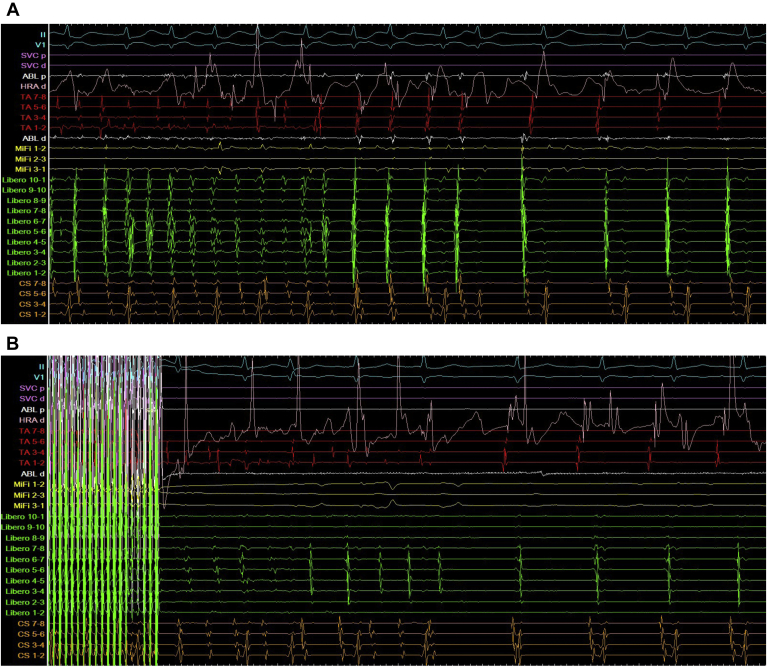
Atrial electrograms during ablation of fractionated signal areas in atrial muscle (FAAM) in left atrium. **A:** Atrial fibrillation (AF) was terminated by ablation on FAAM. **B:** After ablating of FAAM in bilateral atrium, we induced sustained AF via rapid atrial pacing (50 ms/30 mA/5 s) at the high right atrium during isoproterenol infusion, and AF spontaneously returned to sinus.

**Figure 3:**
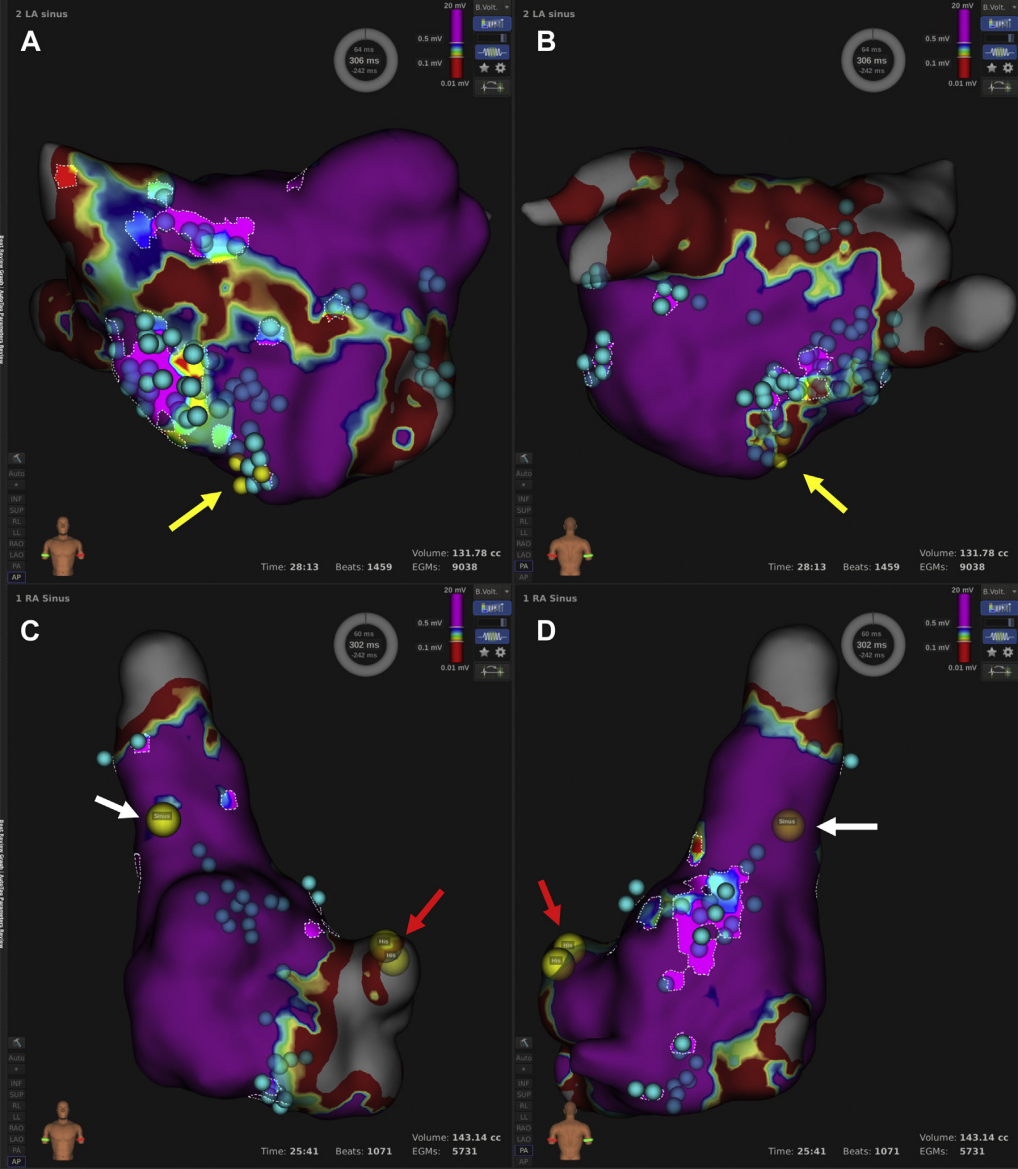
Fractionated signal areas in atrial muscle (FAAM) map and ablation points. FAAM of peaks slider 6.0 were highlighted in left atrium (**A, B**) and right atrium (**C, D**). Blue points show ablation points within the FAAM ablated. Non–pulmonary vein foci are indicated by yellow arrows. Sinus node (*white arrows*) and atrioventricular node (*red arrows*) were not located within FAAM of peaks slider 6.0.

Non-PV triggers, which we successfully detected, were located within the LA FAAM of peaks slider 6.0. We initially ablated the LA FAAM of peaks slider 6.0 during AF, and AF returned to sinus rhythm (Figure 2A). We also ablated the right atrial FAAM of peaks slider 6.0 owing to remaining multiple atrial premature beats (Figure 3). We needed to carefully avoid the sinus node and atrioventricular (AV) node because these areas were highlighted as FAAMs. Non-PV triggers were completely eliminated by administering a bolus injection of ATP (60 mg, until sinus arrest) during continuous infusion of ISP (10 μg/kg/min); finally, AF was not induced. We induced sustained AF via rapid atrial pacing (50 ms/30 mA/5 s) at the high right atrium during ISP infusion, and AF spontaneously returned to sinus rhythm (Figure 2B). No sequelae were observed.

After the discharge, there was no AF recurrence after 1-, 3-, 6-, 12-, and 18-month ECG follow-up and 6- and 12-month 24-Holter ECG follow-up. The patient did not experience any palpitation.

## Discussion

Bilateral PVI at the antrum is the preferred treatment strategy for AF.^1^^,^^3^ The complete elimination of the non-PV foci results in a significant improvement in terms of AF recurrence rates compared to those in patients in whom non-PV triggers were not completely eliminated.^4^^,^^5^ This report shows that FAAM ablation was effective in eliminating the non-PV foci in patients with refractory AF. Therefore, AF recurrence was potentially improved.

The FAAM may play a prominent role in AF induction. Abnormal atrial electrograms recorded during sinus rhythm are related to atrial vulnerabilities such as repetitive atrial firing.^7^^,^^8^ When a single extrastimulus is applied to the atrium early in diastole, the local atrial electrogram will widen.^7^ Moreover, repetitive atrial firing and sustained AF tend to be induced by extrastimuli in patients with fractionated signal areas.^8^ We hypothesized that non-PV triggers that would occur within the FAAM are significantly implicated in the induction and perpetuation of AF. Our novel FAAM map, which can be used as a predictor of the non-PV foci, can help completely eliminate the non-PV foci and improve AF recurrence rates.

Notably, both the sinus node and AV node areas present high-fractionated signals as FAAMs. Therefore, close attention is needed to avoid iatrogenic sick sinus syndrome and AV block.

In this case, FAAM ablation was significantly effective for refractory AF. Non-PV triggers could not be identified by ISP and ATP infusion, and AF was terminated during FAAM ablation. FAAM ablation succeeded in elimination of non-PV triggers. We also performed FAAM ablation in 5 other patients with refractory AF; non-PV triggers were completely ablated successfully (Table 1). They did not experience AF recurrence on regular ECG and 24-Holter ECG follow-up over 1 year.

**Table 1:** Characteristics of other 5 patients who underwent ablation of fractionated signal areas in atrial muscle

Age	Type of rhythm in admission	Previous procedure	Non-PV triggers	Peaks slider of ablating area	Complication	Outcome
68	AF	4 PVI + roof line + LA posterior area	Incessant AF from RA posterior FAAM and multiple APCs from LA	LA 6.0 area and RA 6.0 area	None	Good. No AT/AF recurrence
63	Sinus	4 PVI + CTI	Multiple APCs from bilateral antrum	LA 6.0 area and RA 7.0 area	None	Good. No AT/AF recurrence
66	AF	4 PVI + box isolation + Marshall ablation + posterior mitral isthmus line	Incessant AF from LA bottom FAAM and multiple APCs from RA	LA 5.0 area and RA 5.0 area	None	Good. No AT/AF recurrence by pacemaker remote monitoring
79	AF	4 PVI + CTI	Multiple APCs from bilateral antrum	LA 7.0 area and RA 6.5 area	None	Good. No AT/AF recurrence
47	Sinus	4 PVI + LA/RA septal area + LA posterior area	Incessant AF from RA posterior FAAM	LA 6.0 area and RA 5.0 area	None	Good. No AT/AF recurrence

AF = atrial fibrillation; APC = atrial premature contraction; AT = atrial tachycardia; CTI = cavotricuspid isthmus line; FAAM = fractionated signal areas in atrial muscle; LA = left atrial; PV = pulmonary vein; PVI = pulmonary vein isolation; RA = right atrial.

### Limitations

Although this report is limited by its single-center setting and a small sample size, to the best of our knowledge, this is the first-in-man feasibility study of FAAM ablation. Further investigation is required to validate our findings and to prospectively evaluate the long-term effectiveness of FAAM ablation.

## Conclusion

Our proof-of-concept study demonstrated that the novel FAAM ablation technique successfully eliminates non-PV triggers and improves refractory AF. Larger confirmatory studies are needed to validate that FAAM ablation improves the AF recurrence rate in patients with refractory AF.

**Key Teaching Points**

• Fractionated signal area in the atrial muscle (FAAM) is a valid predictor of the non–pulmonary vein foci in patients with atrial fibrillation (AF).
• Careful attention is needed during FAAM ablation because the sinus node and atrioventricular node also show high FAAM.
• FAAM ablation could improve the recurrence rate of AF in patients with refractory AF. A larger confirmatory study is needed.
